# A Biosensor-Based Quantitative Analysis System of Major Active Ingredients in *Lonicera japonica* Thunb. Using UPLC-QDa and Chemometric Analysis

**DOI:** 10.3390/molecules24091787

**Published:** 2019-05-08

**Authors:** Lin Yang, Hai Jiang, Xudong Xing, Meiling Yan, Xinyue Guo, Wenjing Man, Ajiao Hou, Liu Yang

**Affiliations:** Key Laboratory of Chinese Materia Medica; Heilongjiang University of Chinese Medicine, Ministry of Education, Harbin 150040, China; yanglin0716@126.com (L.Y.); jianghai_777@126.com (H.J.); mrxing_xudong@126.com (X.X.); hxk_yan@163.com (M.Y.); m17645028606@163.com (X.G.); mm1532326@163.com (W.M.); Hou_Ajiao@163.com (A.H.)

**Keywords:** surface plasmon resonance, single mass spectrometry, quantitative analysis, *Lonicera japonica* Thunb., chemometric analysis

## Abstract

In the study, a surface plasmon resonance-based (SPR-based) competitive assay was performed to analyze different compounds’ inhibitory activity to TNF-α, an important pro-inflammatory cytokine in the pathogenesis of chronic inflammatory diseases. Moreover, the single mass spectrometry (MS) detection method was coupled with an ultra-high-performance liquid chromatography (UPLC) system for the routine quality control (QC) of a traditional Chinese medicine (TCM). The above quality control strategy was evaluated with *Lonicera japonica* Thunb. Analytes were firstly separated on a Waters ACQUITYTM UPLC HSS T3 column (2.1 × 50 mm; particle size = 1.8 μm) using a 0.1% formic acid gradient elution, then detected by negative ESI mass spectrometry. The limits of quantification (LOQ) for analytes reached 0.005–0.56 μg/mL. The LOD of the QDa detector was lower than that of the PDA detector, indicating its wider detection range. The QDa detector was also more suitable for the analysis of the complex matrix of TCM. The method showed excellent linearity, with regression coefficients higher than 0.9991. The average recoveries of the investigated analytes were in the range of 98.78–105.13%, with an RSD below 3.91%. The inter-day precision range (*n* = 3 days) was 2.51–4.54%. Compared to other detectors, this strategy could be widely applied in the quantitative analysis of TCM. In addition, the chemically latent data could be revealed using chemometric analysis. Importantly, this study provides an efficient screening method for small-molecule inhibitors targeting the TNF-α pathway.

## 1. Introduction

Traditional Chinese medicines (TCMs) are widely applied in the clinic as complex mixtures of many small-molecule compounds. Appropriate screening methods of small-molecule inhibitors in TCM are not available [1]. A surface plasmon resonance (SPR) biosensor is a technology to monitor the interactions between biomolecules [2]. The past few years have witnessed an increasing interest in applying SPR biosensors during different steps of the drug discovery process, including drug screening [3], and lead compound discovery [4]. Therefore, the SPR technique has great application potential in the analysis of small molecules [5]. The determination methods of the ingredients of TCM in vivo or in vitro are mature [6,7]. Various chromatographic methods have been developed for the quantitative analysis and characterization of TCMs. Among them, some methods, including high-performance liquid chromatography (HPLC)–ultraviolet (UV) [8,9,10,11] and liquid chromatography (LC)–tandem mass spectrometry (MS/MS) [12,13] have been applied in the detection of active ingredients in TCMs. However, since some natural product compounds lack the chromophores required for UV detection, they cannot be directly detected using UV. However, mass spectrometry can be used to identify a certain peak of a compound in different chromatograms. LC–mass spectrometry (LC–MS) is now a routine technique and increasingly available in laboratories [14]. Moreover, it can produce a robust mass spectrometer (MS) signal on the MS detector. Additionally, advanced MS instruments may have higher sensitivity and selectivity, but they are expensive for routine analyses [15,16,17]. Inexpensive and small MS detectors have been developed for LC [18]. Therefore, it is necessary to develop a rapid, sensitive, simple, and small-footprint approach for the quantitative analysis of TCMs.

*Lonicera japonica* Thunb., as a unique TCM widely planted in various provinces in China. *Lonicera japonica* Thunb. (Jinyinhua in Chinese) is listed in the Chinese Pharmacopoeia (2015 Edition) [19]. It was classified into the Caprifoliaceae family and was originally recorded in the “Handbook of Prescriptions for Emergency” [20]. *Lonicera japonica* Thunb. exhibits a broad range of functions such as antioxidant [21], anti-inflammatory [22], anticancer, and anti-carcinogenic activities [23]. Phenolic acids and flavonoids are the major active components in *Lonicera japonica* Thunb. [24]. The contents of active ingredients of *Lonicera japonica* Thunb. vary with planting region. The determination of active ingredients is therefore important in the quality evaluation of *Lonicera japonica* Thunb. [25]. *Lonicera japonica* Thunb. is especially clinically used as an anti-inflammatory. TNF-α is a pro-inflammatory cytokine and is important in the pathogenesis of chronic immune-mediated diseases. TNF-α inhibitors can be used to treat chronic immune-mediated diseases [26]. In recent years, the inhibitors of the TNF-α immune checkpoint pathway have been extensively studied for the treatment of cancers and ischemic stroke [27,28,29]. Chlorogenic acid (3-CQA) is the major active components in *Lonicera japonica* Thunb. [30] and is used as an indicator component of *Lonicera japonica* Thunb. in the Chinese Pharmacopoeia (2015 Edition). In this manuscript we sought to establish the interactions of the major active components in *Lonicera japonica* Thunb. with TNF-α, therefore, it was necessary to develop a simple method to verify these interactions. Furthermore, due to the increased demand for this plant, the price of *Lonicera japonica* Thunb. has been increasing, and some herbal flower buds of species related to *Lonicera* are mistakenly treated as *Lonicera japonica* Thunb. because of their similar morphological characteristics. Therefore, it is necessary to develop an efficient method to quantify active compounds and evaluate the quality of *Lonicera japonica* Thunb. However, compounds with similar polarities cannot be separated from each other or quantified within a short time. For example, for luteoloside and chlorogenic acid, which are mentioned as important indicators of *Lonicera japonica* Thunb. in the Chinese Pharmacopoeia (2015 Edition), it is difficult to achieve baseline separation because they are similar in polarity to other compounds in the complex matrix. These methods require a time-consuming separation process [31,32]. Hence, a single MS detector was coupled to ultra-high-performance liquid chromatography (UPLC) to solve these problems.

In this work, the competitive binding effects between 3-CQA and TNF-a were explored with SPR technology. The binding affinity of 3-CQA was measured. The study is important for screening TNF-α inhibitors in inflammatory immunotherapy. Moreover, representative compounds in *Lonicera japonica* Thunb. were selected to establish a new method for the analysis and quantification of the TCM. A single quad MS detector was applied to quantify ingredients of *Lonicera japonica* Thunb. for the first time. In addition, we compared the advantages of PDA detectors and single MS detectors in the quality control of the TCM. Moreover, we validated the developed method in terms of specificity, precision, stability, and recovery based on International Conference on Harmonization (ICH) guidelines [33]. The chemically latent data were acquired using chemometric analysis.

## 2. Results and Discussion

### 2.1. Preparation of the Sensor Chip Surface

**Figure 1:**
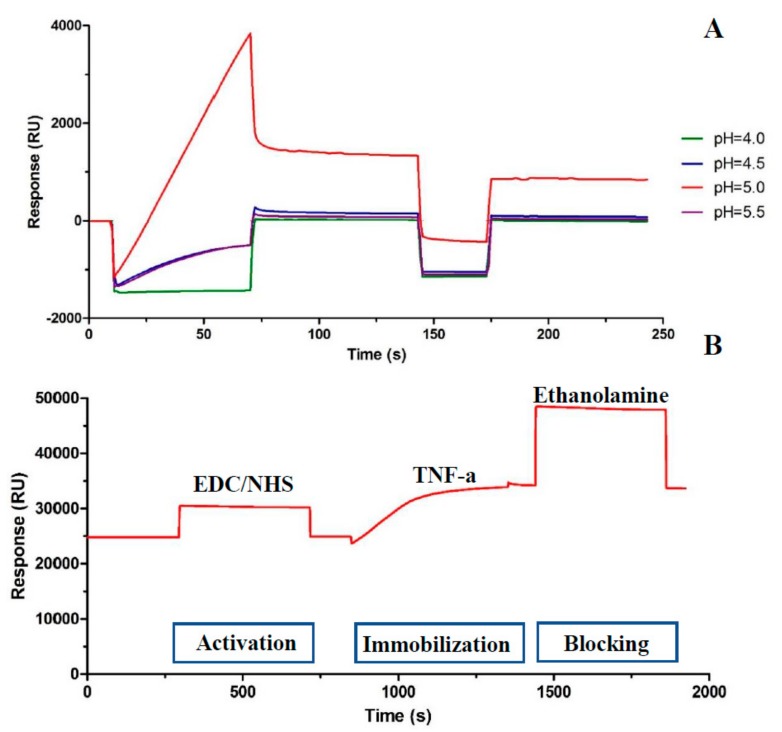
(**A**) Response of immobilization assay at different pH levels. (**B**) Amino coupling of TNF-a with the wizard model.

The CM5 sensor chip surface was first prepared. In order to determine the optimal isoelectric point of the protein, the acetate was tested at various pH values. A high immobilization level in the acetate (pH of 5.0) was realized (Figure 1A). The acetate (i.e., for pH levels of 4.0, 4.5, and 5.5) was selected as the dilution buffer for TNF-α in the immobilization assay. The primary amine groups of TNF-α spontaneously reacted with reactive succinimide esters, which were activated by the mixture of N-hydroxysuccinimide (NHS) and 1-ethyl-3-(3-dimethylaminopropyl) carbodiimide hydro-chloride (EDC). Then, ethanolamine was added to deactivate the excess reactive groups. The immobilization level was 7986.45 RU for flow cell 2 (Figure 1B).

### 2.2. Affinity of 3-CQA Inhibitors to TNF-α

**Figure 2:**
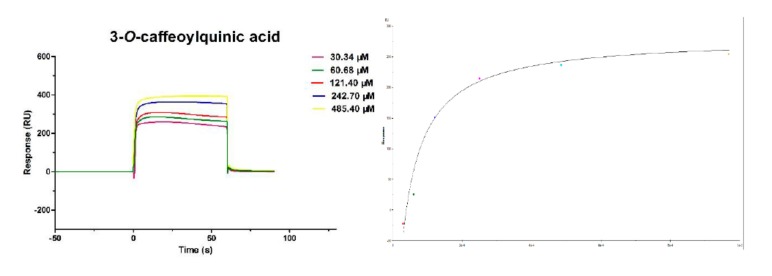
Affinity between 3-CQA and TNF-a. A series of concentrations (30.34–485.40 μM) of 3-CQA were tested to obtain the affinity between TNF-α and 3-CQA by kinetic analysis. The KD value of the interaction between TNF-α and 3-CQA was determined to be 1.38 × 10^−6^ M (*n* = 3).

3-CQA was diluted in water to obtain a series of concentrations (i.e., 30.34–485.40 μM). The affinity of 3-CQA (Figure 2) to TNF-α was assessed using the Biacore T200 Evaluation Software 3.0. The assessment results showed that 3-CQA could interact with TNF-α. The equilibrium dissociation constant (KD) was used to evaluate the binding activity of each compound. The KD value for the interaction between TNF-α and 3-CQA was 1.38 × 10^−6^ M (*n* = 3). This indicates that 3-CQA has a strong binding capacity with TNF-α, which may be one of the main reasons for the anti-inflammatory effect of 3-CQA. Proteins must be modified before the assessment, so the screening procedure was complicated. SPR avoids the modification step and can realize the real-time monitoring of intermolecular interactions. Although SPR can efficiently evaluate the binding affinities of small molecules, other cellular and animal-based assays should also be used to further validate the efficacies of small molecules in vitro and in vivo. Therefore, we established a validation method in the manuscript from the interaction of the major active components in *Lonicera japonica* Thunb. with TNF-α. It is important to provide a theoretical basis for the data on active ingredients in *Lonicera japonica* Thunb. In the future, we will study the regulation between 3-CQA and TNF-α.

### 2.3. Optimization of Chromatography and Comparison of Detection Conditions

A rapid and comprehensive analysis method is required for the fast analysis of the components in *Lonicera japonica* Thunb. The simultaneous determination of 11 analytes (including the internal standard, IS) was performed with the UPLC–PDA–QDa system, then modified appropriately for determining the samples.

**Figure 3:**
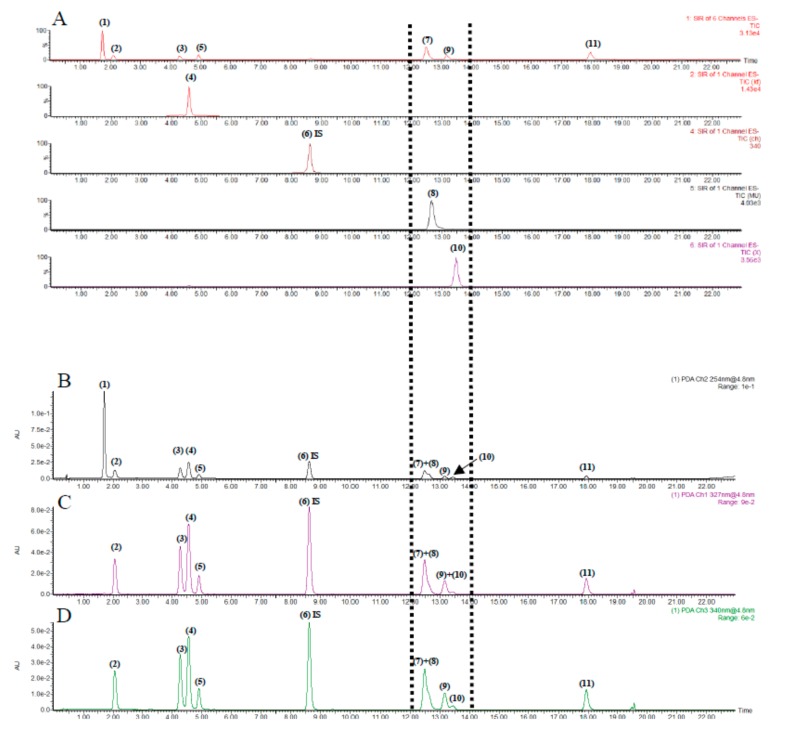
(**A**) Representative extraction ion chromatograms of the samples with IS and chromatograms of samples with IS determined at different wavelengths of (**B**) 254, (**C**) 327, and (**D**) 340 nm. The mixed reference standards of (**1**) 3,4-dihydroxybenzoic acid, (**2**) 5-O-caffeoylquinic acid, (**3**) 3-O-caffeoylquinic acid, (**4**) caffeic acid, (**5**) 4-*O*-caffeoylquinic acid, (**6**) IS (chloramphenicol), (**7**) 3,5-*O*-di-caffeoylquinic acid, (**8**) luteoloside, (**10**) rutin, (**9**) 3,4-*O*-di-caffeoylquinic acid, and (**11**) 4,5-*O*-di-caffeoylquinic acid.

To realize a good separation effect and high sensitivity, the UPLC–PDA method was optimized firstly for the systematic analysis of all analytes. Then chromatographic conditions, such as the additive in the mobile phase, the stationary phase, flow rate, column temperature, and different wavelengths, were analyzed. Among the three tested columns (HSS T3, BEH C18, and Thermo Hypersil GOLD), the HSS T3 column yielded a good peak shape. However, 3,5-*O*-dicaffeoylquinic acid (**7**) and luteoloside (**8**) did not achieve an effective baseline separation on the PDA detector, even at the optimal absorption wavelength of each analyte within a short time (Figure 3B–D). The same result was obtained in the separation of 3,4-*O*-dicaffeoylquinic acid (**9**) and rutin (**10**). Luteoloside is another important indicator of *Lonicera japonica* Thunb. in the Chinese Pharmacopoeia (2015 Edition). Compared to PDA detectors, QDa detectors can be applied in the analysis by detecting molecular weights in a short period (Figure 3A). For targeted analyses and a targeted assay, analysis data can be collected simply by using the SIR scanning mode, thus greatly simplifying the analysis and increasing S/N and sensitivity. The structure of the compound and the MS information in negative ion mode are shown in Figure S1. The separation of phenolic acid and flavonoids by using QDa detectors were not reported. Thus, we further improved the analysis and separation of phenolic acid and flavonoids in *Lonicera japonica* Thunb. by using QDa detectors.

**Figure 4:**
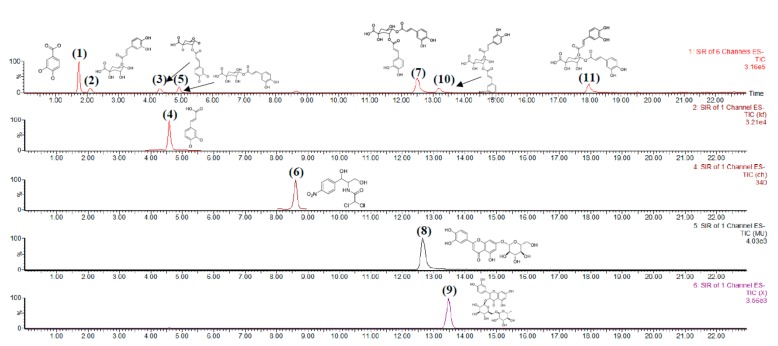
Extraction ion chromatograms of samples with IS of (**1**) 3,4-dihydroxybenzoic acid, (**2**) 5-*O*-caffeoylquinic acid, (**3**) 3-*O*-caffeoylquinic acid, (**4**) caffeic acid, (**5**) 4-*O*-caffeoylquinic acid, (**6**) IS (chloramphenicol), (**7**) 3,5-*O*-di-caffeoylquinic acid, (**8**) luteoloside, (**9**) rutin, (**10**) 3,4-*O*-di-caffeoylquinic acid, and (**11**) 4,5-*O*-di-caffeoylquinic acid.

Figure 4 shows the chromatograms of *Lonicera japonica* Thunb. obtained with QDa detectors. Table 1 shows the comparison of previous methods and the new method for the analysis of 10 compounds in a short time (less than 20 min). Furthermore, we compared the precision and detection limits of PDA and QDa detectors to verify the new method.

### 2.4. Method Validation

The proposed UPLC–PDA–QDa method was verified by determining the linearity, limit of quantification (LOQ), limit of detection (LOD), stability, precision, and recovery.

#### 2.4.1. Linearity, LOD, and LOQ

The calibration curves of QDa detectors were obtained with a peak area ratio (i.e., analyte/IS) versus the analyte concentration. Linear regression equations and correlation coefficients for all analytes showed good linearity (Table 2; Table 3). Correlation coefficients (R2) ranged from 0.9991 to 0.9999. The signal-to-noise ratios of LOD and LOQ were 3 and 10, respectively. The LODs and LOQs of QDa were 0.001–0.17 μg/mL and 0.005–0.56 μg/mL, respectively. The QDa detector has a lower LOD than the PDA detector, indicating its wider detection range. The QDa detector is more suitable for analyzing TCM.

#### 2.4.2. Stability, Precision, and Recoveries

Inter-day and intra-day precisions were examined with each analyte in six replicates in a single day and in triplicate for three consecutive days. The validation results of the UPLC–PDA and UPLC–QDa systems are provided in Table 2 and Table 3. The RSDs of the intra-day and inter-day precisions of the two detectors are in acceptable limits. The RSDs of stability for the analytes using UPLC–PDA and UPLC–QDa were below 2.99%. Average recoveries of the investigated analytes were in the range of 98.78–105.13%, with RSDs below 3.91%.

However, the QDa detector is better than the PDA detector in the quantification of *Lonicera japonica* Thunb, because it can identify a certain peak in different chromatograms based on the molecular mass of compounds due to its high specificity and reduced analysis time.

In summary, the developed UPLC–QDa detector could yield satisfactory results for the simultaneous determination of 11 analytes in *Lonicera japonica* Thunb. In this work, we chose the UPLC–QDa detector to determine the content of *Lonicera japonica* Thunb. from different regions.

### 2.5. Quantitative and Boxplot Analysis

**Figure 5:**
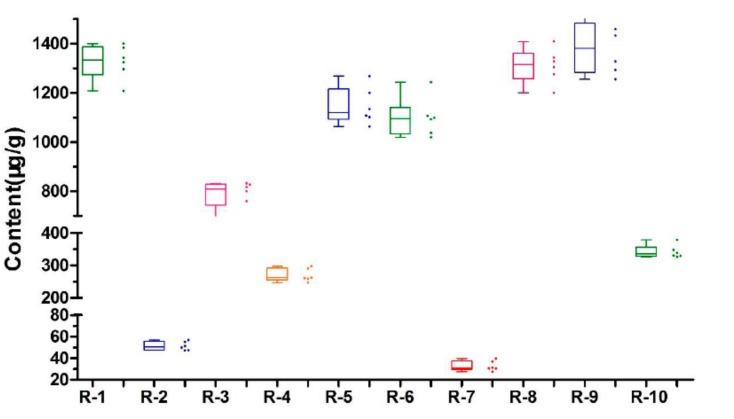
Boxplots of luteoloside in *Lonicera japonica* Thunb. from different regions. * Indicates the strands of the Chinese Pharmacopoeia (2015 Edition).

The 10 analytes in 10 batches of *Lonicera japonica* Thunb. samples collected from different locations in China were analyzed with the UPLC–QDa method. The contents of 10 selected compounds were calculated with calibration curves according to the IS method (Table 2). Each sample was analyzed in sextuplicate and the mean content was obtained. The contents of 10 compounds in *Lonicera japonica* Thunb. from various geographical origins showed significant differences (Table 4). The highest content of luteoloside was obtained in the Guangdong sample R9 (1387.61 ± 115.22 μg/g). The box plots (Figure 5) show the levels of luteoloside in *Lonicera japonica* Thunb. All the data are presented as box plots with medians. The content of luteoloside in the samples did not meet the requirements of the Chinese Pharmacopoeia (2015 Edition). These unqualified samples may have adulterated or unqualified flower buds. Meanwhile, the contents of chlorogenic acid in the samples from each geographical origin met the requirements of the Chinese Pharmacopoeia (2015 Edition).

### 2.6. Chemometric Analysis

Nowadays, chemometric analysis has been extensively applied in the determination of origins and chemical markers of TCMs [16,37]. Therefore, chemometric methods can reveal the differences in medicinal materials from different producing areas.

#### Evaluation by Hierarchical Cluster Analysis (HCA)

HCA can find homogeneous clusters of cases from measured characteristics [38]. Samples are clustered into different groups based on similarity. The peak areas of 10 components in the QDa fingerprints were defined as the variables in the analysis in order to differentiate the 10 batches of samples of *Lonicera japonica* Thunb. (Figure 3).

**Figure 6:**
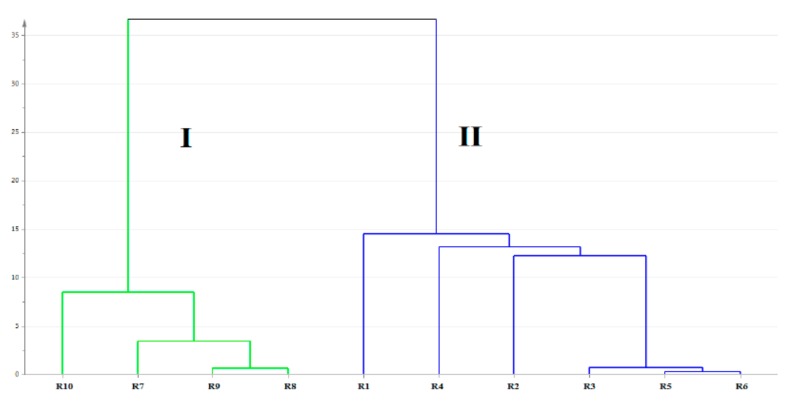
Hierarchical cluster analysis (HCA) dendrograms of different samples of *Lonicera japonica* Thunb. I Group 1; II Group 2.

The variances among clusters were analyzed using Ward’s method. Two dendrograms were obtained (Figure 6) to display the relationships among different samples. The 10 batches of samples of *Lonicera japonica* Thunb. (Figure 3) could be obviously classified into Clusters I and II. The HCA results could more intuitively and simply distinguish the samples of *Lonicera japonica* Thunb. from various origins.

## 3. Materials and Methods

### 3.1. Chemicals, Reagents, and Materials

Chemicals and reagents used in the experiment are listed in Table 5. The 10 batches of samples of *Lonicera japonica* Thunb. were obtained from various regions in China. Professor Su Lianjie from Heilongjiang University of Chinese Medicine, Harbin, China identified all the samples as *Lonicera japonica* Thunb.

### 3.2. Instrumentation

SPR experiments were carried out at 25 °C by a Biacore T200 system (GE Healthcare, Uppsala, Sweden). PBS-P buffer was filtered through a 0.45-μm membrane and degassed before use. After the surface of the CM5 sensor chip was prepared, TNF-α was immobilized on the surface according to the following procedure. Sodium acetate solution was used to prepare 20 μg/mL of TNF-a at a pH of 5.0. The immobilization pH was determined using the protein isoelectric points described in Section 2.1. With the Amine Coupling Kit, diluted TNF-α was immediately immobilized onto a flow cell of the CM5 sensor chip.

The UPLC-MS analyses were performed on an ACQUITY UPLC system equipped with a photo-diode array (PDA) detector (Waters, Milford, MA, USA) coupled to a mass single-quadrupole detector (QDa Waters), which is a compact detector with an electrospray ionization (ESI) interface. MassLynx V4.1 software was used for instrument control and data acquisition. Samples were separated on an HSS T3 column (50 × 2.1 mm, 1.8 μm. Waters). The mobile phase comprised a mixture of methanol and 0.1% *v*/*v* formic acid. The elution procedure was set as follows: 0–5 min at 10–20% methanol, 5–20 min at 20–90% methanol, and 20–22 min at 100% methanol. The system was re-equilibrated for 3.0 min with 10% methanol eluent for the next injection. Under the flow rate of the mobile phase (0.3 mL/min), good nebulization efficiency was realized. Column temperature and sample injection volume were respectively set to be 45 °C and 2 μL. With the PDA detector, phenolic acid was monitored at 327 and 254 nm, and the flavonoid was monitored at 340 nm (for 13–30 min). The single ion recording (SIR) method was established on a single quadruple MS detector. The QDa conditions were set as follows: a cone voltage of 20 V, a capillary voltage of 0.8 kV, a desolvation gas flow of 800 L·h−1, and a source temperature of 600 °C. The data were acquired under the SIR mode. The total run time was 20 min.

### 3.3. Immobilization of TNF-α on a SPR Sensor

A Biacore T200 system was used to carry out SPR assays. Channel 1 was selected as the reference channel and Channel 2 was selected as the detection channel. Recombinant TNF-a was first diluted in 10 mM acetate (pH of 5.0 or 20 μg/mL) and then immobilized on channel cells via a EDC/NHS cross-linking reaction. Finally, ethanolamine was used to block the activated channels.

### 3.4. Interactions between Small Molecules and TNF-α

Various small molecules were diluted in PBS-P buffer according to a series of concentrations (30.34–485.40 μM for mono 3-CQA) and flowed over immobilized TNF-α at a flow rate of 30 μL/min. The contact time and dissociation time was respectively set to, 60 and 150 s, respectively. A flow cell without a coupling protein was used as the blank reference. Blank solutions without analyte were adopted to correct system errors. The binding activity of small molecules was evaluated with the equilibrium dissociation constant (KD). Experimental data were analyzed using a Biacore T200 instrument.

### 3.5. Preparation of Samples

Air-dried materials were first ground and passed through a 60-mesh sieve. An aliquot of 0.5 g of powder was extracted with 20 mL of 50% methanol using 30-min ultrasonic extraction. After the extracts were cooled to room temperature and the same volume of solvent was added, the solution was centrifuged (16,000 rpm for 5 min). The obtained supernatant was filtered through 0.22-μm membrane prior to injection.

### 3.6. Standard Solution Preparation

Appropriate amounts of 11 standards were dissolved in methanol to prepare stock solutions, which were diluted with methanol for chromatographic analysis. Standard solutions were stored at 4 °C until use and filtered through a 0.22-μm membrane before injecting them into the UPLC–PDA–QDa system.

### 3.7. Method Validation

The obtained data and analytical method were validated according to the requirements of the routine quality control (QC) analysis. The specificity of the LC–QDa method was validated according to the regulatory guidelines of ICH [20] and the Guidance of Good Manufacturing Practices for Drugs (CFDA) [24]. Specificity, precision, stability, LOQ, and LOQ were also evaluated.

### 3.8. Data Analysis

The experiments were carried out in triplicate at least. Experimental data were expressed as mean ± RSD. Differences among various groups were believed to be significant at *p* < 0.05. The HCA of the samples of *Lonicera japonica* Thunb. were used for qualitative analysis in SIMCA-P 13.0 (Umetrics, San Jose, CA, USA).

## 4. Conclusions

It is necessary to develop appropriate methods for screening small-molecule inhibitors. This study provides a new and efficient method for evaluating 3-CQA inhibitors targeting the TNF-α pathway in TCM. We developed a simple, fast and robust method for the QC analysis of active ingredients in TCM. This method met the requirements of the ICH-Q2 and has been successfully applied in the routine QC analysis of *Lonicera japonica* Thunb. The LOD and LOQ values for each analyte were low enough to determine the contents of these analytes in the samples of *Lonicera japonica* Thunb, and these were comparable to values given in previous reports. Moreover, the established method offers a revolutionary framework for the QC of compounds in TCM. Importantly, this study is the first report on the QC analysis of TCM by UPLC–QDa. This validated assay will be further applied for the analysis of TCM or complex matrices. In summary, the quantitative analysis method combined with SPR technology can improve the quality control of TCM.

## Figures and Tables

**Table 1:** Overview of the mentioned analysis methods of *Lonicera japonica* Thunb.

Separation Methods	Analytes	Stationary Phases	Mobile Phases	T (min)	LOD (μg/mL)	Ref.
HPLC–DAD	10 phenolic acids	AQ-C18 column 4.6 × 250 mm, 5 μm	Methanol and 0.1% aqueous formic acid	55	0.01–0.05	[31]
RP–HPLC–DAD	7 phenolic acids	Agilent C18 4.6 × 250 mm, 5 μm	Acetonitrile and 0.2% aqueous phosphoric acid	60	0.02–1.58	[34]
HPLC–PDA	7 phenolic acids	Luna 5 μm C18 4.6 × 250 mm, 5 μm	Methanol and 0.1% aqueous phosphoric acid	60	0.02–0.08	[35]
HPLC–DA–ELSD	6 phenolic acids	Agilent Zorbax ODS guard column 6.0 × 25 mm, 5 μm	Acetonitrile and 0.4% aqueous *v*/*v* acetic acid	50	0.04–0.17	[36]

**Table 2:** Regression equations, linearity ranges, correlation coefficients, limits of quantitation (LOQ), limits of detection (LOD), and inter-day and intra-day precisions and stability for 12 analytes using the QDa detector.

Peak No.	Analytes	Calibration Curves	R^2^	Linear Ranges (μg/mL)	LOQ (μg/mL)	LOD (μg/mL)	Precisions (%, RSD)		Stability (%, RSD)	Recovery	
							Intra-Day (*n* = 6)	Inter-Day (*n* = 3)		Mean Recovery (%)	RSD (%)
1	3,4-dihydroxybenzoic acid	y = 12.26x + 7.06	0.9991	0.01–22.00	0.005	0.001	2.73	3.19	1.50	104.64	2.21
2	5-*O*-caffeoylquinic acid	y = 1.01x + 0.56	0.9995	1.20–23.50	0.34	0.11	4.80	4.54	2.77	105.13	3.10
3	3-*O*-caffeoylquinic acid	y = 0.57x + 0.77	0.9995	1.56–45.70	0.41	0.13	4.40	4.41	2.99	100.22	3.46
4	caffeic acid	y = 4.17x + 1.57	0.9991	10.5–105.0	0.14	0.04	1.55	3.12	1.80	99.16	2.45
5	4-*O*-caffeoylquinic acid	y = 1.76x + 1.62	0.9991	6.50–65.00	0.06	0.02	1.37	3.68	2.00	102.98	3.58
7	3,5-*O*-dicaffeoylquinic acid	y = 6.43x + 0.66	0.9998	2.35–23.50	0.56	0.17	3.72	3.68	1.77	98.78	3.91
8	luteoloside	y = 0.67x + 1.78	0.9991	3.47–34.70	0.30	0.09	2.26	3.47	2.31	102.77	2.67
9	3,4-*O*-dicaffeoylquinic acid	y = 0.79x + 0.41	0.9992	10.2–145.00	0.50	0.16	2.83	4.10	2.78	102.45	3.23
10	rutin	y = 1.87x − 1.23	0.9991	8.83–22.07	0.19	0.05	1.86	2.92	2.96	99.69	2.44
11	4,5-*O*-dicaffeoylquinic acid	y = 4.24x + 1.00	0.9995	2.02–20.2	0.50	0.15	1.08	2.51	1.76	102.44	2.34

**Table 3:** Regression equations, linearity ranges, correlation coefficients, LOQ, LOD, and inter-day and intra-day precisions and stability for six analytes using the PDA detector.

Peak No.	Analytes	Calibration Curves	R^2^	Linear Ranges (μg/mL)	LOQ (μg/mL)	LOD (μg/mL)	Precisions (%, RSD)		Stability (%, RSD)	Recovery	
							Intra-Day (*n* = 6)	Inter-Day (*n* = 3)		Mean Recovery (%)	RSD (%)
1	3,4-dihydroxybenzoic acid	y = 1287.1x + 2760.3	0.9991	0.10–15.20	0.80	0.26	2.60	2.48	1.44	103.14	2.10
2	5-*O*-caffeoylquinic acid	y = 1487.1x + 3010.8	0.9991	2.26–22.60	1.06	0.34	0.59	3.23	2.74	101.77	3.07
3	3-*O*-caffeoylquinic acid	y = 1485.8x + 174.89	0.9992	1.56–15.60	1.21	0.41	2.00	4.40	2.48	97.24	3.15
4	4-*O*-caffeoylquinic acid	y = 1317.7x + 496.99	0.9999	1.33–13.35	0.06	0.02	1.14	1.93	1.66	101.78	3.38
5	caffeic acid	y = 4782.7x + 319.78	0.9999	0.14–7.05	0.10	0.03	2.94	4.38	1.79	98.44	2.23
11	4,5-*O*-dicaffeoylquinic acid	y = 1612.1x − 424.03	0.9995	2.35–10.15	1.65	0.52	2.89	5.83	1.56	101.74	2.01

**Table 4:** Contents of investigated components in *Lonicera japonica* Thunb.

No.	Areas	Locations (Latitude, Longitude)	Contents of Investigated Components (*n* = 3, μg/g)									
			1	2	3	4	5	7	8	9	10	11
R1	Pingyi, Shandong	35° 51 N, 117° 64 E	46.80 ± 12.33	297.97 ± 41.69	63,147.59 ± 1345.4	79.00 ± 10.46	921.10 ± 49.40	122.44 ± 32.16	1326.08 ± 68.99	49,818.36 ± 232.26	1451.50 ± 50.22	1430.76 ± 40.73
R2	Julu, Hebei	37° 22 N, 115° 04 E	299.48 ± 42.78	226.08 ± 56.91	30,373.36 ± 710.24	168.24 ± 26.39	1082.35 ± 259.47	20.00 ± 6.11	51.45 ± 4.01	46,757.00 ± 1484.57	416.03 ± 14.21	1170.78 ± 337.23
R3	Zhengzhou, Henan	34° 66 N, 114° 08 E	30.94 ± 2.29	110.40 ± 4.80	20,692.08 ± 410.92	46.05 ± 8.72	390.18 ± 76.46	50.33 ± 2.68	789.27 ± 52.15	24,586.97 ± 858.41	1164.39 ± 20.39	847.60 ± 32.43
R4	Weinan, Shanxi	34° 50 N, 109° 45 E	137.23 ± 13.45	271.44 ± 46.16	30,339.48 ± 1603.90	132.63 ± 14.49	791.90 ± 55.23	35.39 ± 4.24	269.55 ± 19.75	62,484.82 ± 713.87	101.30 ± 9.22	1685.51 ± 186.52
R5	Wuhan, Hubei	30° 18 N, 114° 96 E	38.50 ± 9.56	88.43 ± 12.11	24,227.80 ± 1366.86	41.52 ± 14.14	264.83 ± 65.15	68.67 ± 2.56	1145.96 ± 75.00	26,599.83 ± 883.28	1325.01 ± 409.60	717.37 ± 13.78
R6	Shaoyang, Hunan	27° 23 N, 111° 46 E	70.37 ± 17.13	121.53 ± 41.69	25,286.46 ± 558.17	74.62 ± 26.35	474.84 ± 46.04	74.24 ± 19.64	1099.91 ± 79.07	34,091.29 ± 728.19	1053.97 ± 24.82	998.62 ± 23.17
R7	Nanchang, Jiangxi	28° 70 N, 115° 83 E	147.98 ± 34.73	180.17 ± 24.67	26,088.33 ± 796.36	94.34 ± 9.40	693.50 ± 87.45	24.50 ± 3.99	32. 69 ± 4.61	37,265.90 ± 615.11	15,740.65 ± 251.10	888.22 ± 29.96
R8	Baise, Gaungxi	23° 91 N, 106° 60 E	243.42 ± 11.76	492.17 ± 11.14	41,481.32 ± 1295.01	194.94 ± 4.87	1371.60 ± 334.51	64.86 ± 1.10	1310.36 ± 70.01	89,976.03 ± 1060.17	161.14 ± 5.53	2598.15 ± 58.70
R9	Meizhou, Guangdong	24° 33 N, 116° 20 E	159.37 ± 35.83	351.07 ± 79.29	32,244.27 ± 1469.74	181.09 ± 16.96	886.54 ± 105.04	39.18 ± 10.11	1387.61 ± 115.22	59,152.84 ± 1339.82	173.31 ± 25.31	1767.55 ± 550.20
R10	Kunming, Yunnan	24° 76 N, 102° 96 E	25.65 ± 9.54	42.20 ± 4.68	29,870.24 ± 1057.21	89.14 ± 3.55	214.11 ± 27.56	842.08 ± 68.74	342.42 ± 19.64	69,825.26 ± 1012.28	1905.86 ± 210.44	1234.42 ± 21.85

**Table 5:** Experimental chemicals and reagents.

Chemicals and Reagents	Sources
Recombinant human TNF-α protein	Novoprotein (Shanghai, China)
Sensor chips (CM 5)	GE Healthcare Life Science (Uppsala, Sweden)
Immobilization buffer (acetate to pH levels of 5.5, 5.0, 4.5, and 4.0)	
PBS-P buffer (10 mM phosphate buffer containing 137 mM NaCl, 2.7 mM KCl, and 0.05% surfactant P20, with a pH of 7.4)	
Regeneration solutions (10 mM NaOH)	
Amine Coupling Kit (EDC and NHS; 1.0 M ethanolamine (pH of 8.5))	
Glycine 2.0	
Methanol (HPLC grade)	Fisher Scientific (Pittsbargh, PA, USA)
Water	Hangzhou Wahaha group (Hangzhou, China)
Formic acid	Dikma Co. (Richmond Hill, NY, USA)
Internal standards (chloramphenicol) (purity ≥99.0%)	Sigma (St. Louis, MO, USA)
3,4-dihydroxybenzoic acid (purity ≥99.0%)	Chengdu Must Bio-technology Co., Ltd. (Chengdu, China)
Caffeic acid (purity ≥99.0%)	
3-*O*-caffeoylquinic acid (purity ≥99.0%)	
4-*O*-caffeoylquinic acid (purity ≥99.0%)	
5-*O*-caffeoylquinic acid (purity ≥99.0%)	
3,5-*O*-di-caffeoylquinic acid (purity ≥99.0%)	
3,4-*O*-di-caffeoylquinic acid (purity ≥99.0%)	
4,5-*O*-di-caffeoylquinic acid (purity ≥99.0%)	
Rutin (purity ≥ 99.0%)	
Luteoloside (purity ≥ 99.0%)	

## Supplementary Materials

The following are available online, Figure S1.

## References

[1] Han Y., Gao Y., He T., Wang D., Guo N., Zhang X., Wang H. (2018). PD-1/PD-L1 inhibitor screening of caffeoylquinic acid compounds using surface plasmon resonance spectroscopy. Anal. Biochem..

[2] Wang Y.H., Tang J.G., Wang R.R., Yang L.M., Dong Z.J., Du L., Zheng Y.T. (2007). Flazinamide, a novel β-carboline compound with anti-HIV actions. Biochem. Bioph. Res. Co..

[3] Chen L., Cao Y., Zhang H., Lv D., Zhao Y., Liu Y., Chai Y. (2018). Network pharmacology-based strategy for predicting active ingredients and potential targets of Yangxinshi tablet for treating heart failure. J. Ethnopharmacol..

[4] Liu L. (2014). Efficient Hit and Lead Compound Evaluation Strategy Based on Off-Rate Screening by Surface Plasmon Resonance. J. Med. Chem..

[5] Wang S., Dong Y., Liang X. (2018). Development of a SPR aptasensor containing oriented aptamer for direct capture and detection of tetracycline in multiple honey samples. Biosens. Bioelectron..

[6] Yang L., Jiang H., Yan M., Xing X., Guo X., Man W., Kuang H. (2019). Comparison of Pharmacokinetics of Phytoecdysones and Triterpenoid Saponins of Monomer, Crude and Processed Radix *Achyranthis bidentatae* by UHPLC-MS/MS. Xenobiotica.

[7] Li C.R., Li M.N., Yang H., Li P., Gao W. (2018). Rapid characterization of chemical markers for discrimination of Moutan Cortex and its processed products by direct injection-based mass spectrometry profiling and metabolomic method. Phytomedicine.

[8] Zhao X., Kong W., Zhou Y., Wei J., Yang M. (2017). Evaluation and quantitative analysis of 11 compounds in Morinda officinalis using ultra-high performance liquid chromatography and photodiode array detection coupled with chemometrics. J. Sep. Sci..

[9] Jiang H., Yang L., Xing X., Yan M., Guo X., Yang B., Kuang H. (2018). HPLC-PDA Combined with Chemometrics for Quantitation of Active Components and Quality Assessment of Raw and Processed Fruits of *Xanthium strumarium* L.. Molecules.

[10] Jiang H., Yang L., Xing X., Yan M., Guo X., Yang B., Kuang H.X. (2018). Development of an analytical method for separation of phenolic acids by ultra-performance convergence chromatography (UPC^2^) using a column packed with a sub-2-μm particle. J. Pharm. Biomed. Anal..

[11] Xing X., Yan M., Zhang X., Yang L., Jiang H. (2017). Quantitative analysis of triterpenoids in different parts of *Aralia elata* (Miq.) Seem using HPLC–ELSD and their inhibition of human umbilical vein endothelial cell ox-LDL-induced apoptosis. J. Liq. Chromatogr. Relat. Technol..

[12] Shu Z., Li X., Rahman K., Qin L., Zheng C. (2016). Chemical fingerprint and quantitative analysis for the quality evaluation of Vitex negundo seeds by reversed-phase high-performance liquid chromatography coupled with hierarchical clustering analysis. J. Sep. Sci..

[13] Gul W., Gul S.W., Chandra S., Lata H., Ibrahim E.A., El Sohly M.A. (2018). Detection and Quantification of Cannabinoids in Extracts of Cannabis sativa Roots Using LC-MS/MS. Planta Med..

[14] Kormány R., Molnár I., Fekete J. (2017). Renewal of an old European Pharmacopoeia method for Terazosin using modeling with mass spectrometric peak tracking. J. Pharm. Biomed. Anal..

[15] Xing J., Zang M., Zhang H., Zhu M. (2015). The application of high-resolution mass spectrometry-based data-mining tools in tandem to metabolite profiling of a triple drug combination in humans. Anal. Chim. Acta.

[16] Yang L., Jiang H., Yan M., Xing X., Guo X., Yang B., Kuang H. (2018). UHPLC-MS/MS Quantification Combined with Chemometrics for Comparative Analysis of Different Batches of Raw, Wine-Processed, and Salt-Processed Radix *Achyranthis bidentatae*. Molecules.

[17] Holčapek M., Kolářová L., Nobilis M. (2008). High-performance liquid chromatography–tandem mass spectrometry in the identification and determination of phase I and phase II drug metabolites. Anal. Bioanal. Chem..

[18] Bu X., Regalado E.L., Hamilton S.E., Welch C.J. (2016). The emergence of low-cost compact mass spectrometry detectors for chromatographic analysis. TrAC Trends Anal. Chem..

[19] Chinese Pharmacopoeia Commission (2015). Pharmacopoeia of People’s Republic of China.

[20] Wang Y., Xiao C., Tian L. (2010). Disscussion on herbal textual research on *Flos lonicerae*. China J. Chin. Mater. Med..

[21] Seo O.N., Kim G.S., Park S., Lee J.H., Kim Y.H., Lee W.S., Shin S.C. (2012). Determination of polyphenol components of Lonicera japonica Thunb. using liquid chromatography–tandem mass spectrometry: Contribution to the overall antioxidant activity. Food Chem..

[22] Kang M., Jung I., Hur J., Kim S.H., Lee J.H., Kang J.Y., Lee J.D. (2010). The analgesic and anti-inflammatory effect of WIN-34B, a new herbal formula for osteoarthritis composed of *Lonicera japonica* Thunb. and *Anemarrhena asphodeloides* BUNGE in vivo. J. Ethnopharmacol..

[23] Cai Y., Luo Q., Sun M., Corke H. (2004). Antioxidant activity and phenolic compounds of 112 traditional Chinese medicinal plants associated with anticancer. Life Sci..

[24] Peng Y., Liu F., Ye J. (2010). Determination of Phenolic Acids and Flavones in *Lonicera japonica* Thumb. by Capillary Electrophoresis with Electrochemical Detection. J. Electroanal. Chem..

[25] Zhang D.Y., Yao X.H., Duan M.H., Wei F.Y., Wu G.H., Li L. (2015). Variation of essential oil content and antioxidant activity of Lonicera species in different sites of China. Ind. Crops Prod..

[26] Murdaca G., Spanò F., Contatore M., Guastalla A., Penza E., Magnani O., Puppo F. (2015). Infection risk associated with anti-TNF-α agents: A review. Expert Opin. Drug. Saf..

[27] Wang C.Y., Mayo M.W., Baldwin A.S. (1996). TNF-and cancer therapy-induced apoptosis: Potentiation by inhibition of NF-κB. Science.

[28] Cemazar M., Todorovic V., Scancar J., Lampreht U., Stimac M., Kamensek U., Sersa G. (2015). Adjuvant TNF-α therapy to electrochemotherapy with intravenous cisplatin in murine sarcoma exerts synergistic antitumor effectiveness. Radiol. Oncol..

[29] Gu L. (2015). TNF-a (−238 G/A and −308 G/A) gene polymorphisms may not contribute to the risk of ischemic stroke. Int. J. Neurosci..

[30] Zhang B., Yang R., Liu C.Z. (2008). Microwave-assisted extraction of chlorogenic acid from flower buds of Lonicera japonica Thunb. Sep. Purif. Technol..

[31] Kuixia C., Yingtao Z., Xiuwei Y. (2013). Simultaneous quantification of ten phenolic acids in *Lonicerae Japonicae* Flos by HPLC-DAD. J. Chin. Pharm. Sci..

[32] Zhao Y., Dou D., Guo Y., Qi Y., Li J., Jia D. (2018). Comparison of the Trace Elements and Active Components of *Lonicera japonica* flos and Lonicera flos Using ICP-MS and HPLC-PDA. Biol. Trace Elem. Res..

[33] Guideline I.H.T. Validation of analytical procedures: Text and methodology Q2 (R1). Proceedings of the International Conference on Harmonization.

[34] Chunqiu W., Wenxia L. (2015). Simultaneous determination of ten chemical constituents in *Lonicera japonica* Thunb. by RP-HPLC. Chin. Trad. Pat. Med..

[35] Miao L., Yongxiong W. (2014). Determination of eight components in *Lonicerae japonicae* Flos by HPLC. Chin. Trad. Herbal Drugs.

[36] Chen C.Y., Qi L.W., Li H.J., Li P., Yi L., Ma H.L., Tang D. (2015). Simultaneous determination of iridoids, phenolic acids, flavonoids, and saponins in Flos *Lonicerae* and Flos *Lonicerae japonicae* by HPLC-DAD-ELSD coupled with principal component analysis. J. Sep. Sci..

[37] Jiang H., Yang L., Xing X., Yan M., Guo X., Yang B., Kuang H.X. (2019). Chemometrics coupled with UPLC-MS/MS for simultaneous analysis of markers in the raw and processed Fructus Xanthii, and application to optimization of processing method by BBD design. Phytomedicine.

[38] Bridges C.C. (1966). Hierarchical cluster analysis. Psychol. Rep..

