# Supplementary material for: A Biosensor-Based Quantitative Analysis System of Major Active Ingredients in Lonicera japonica Thunb. Using UPLC-QDa and Chemometric Analysis
Source: Molecules. 2019 May 8;24(9):1787. doi: 10.3390/molecules24091787 (PMC6540269; doi:10.3390/molecules24091787)
Supplement: Supplementary file 1 [file molecules-24-01787-s001.pdf]

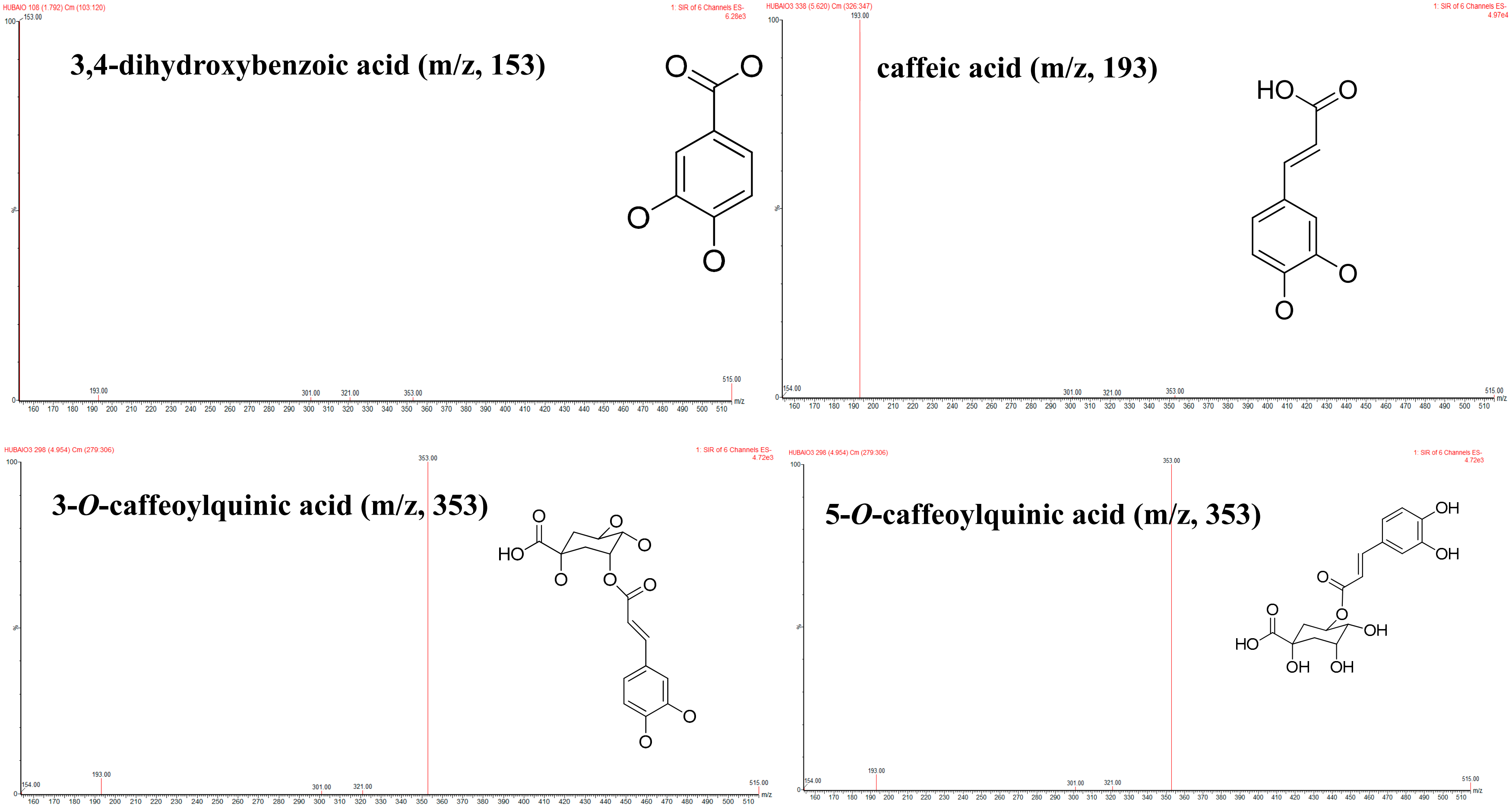

3,4-dihydroxybenzoic acid (m/z, 153)
caffeic acid (m/z, 193)
3-O-caffeoylquinic acid (m/z, 353)
5-O-caffeoylquinic acid (m/z, 353)
HO
OH
m/z
100
0
153.00
193.00
301.00
321.00
353.00
515.00
154.00

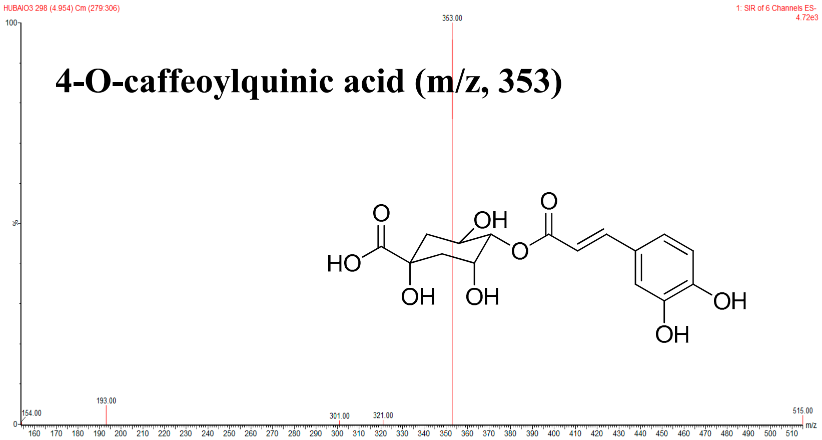
HUBAIO3 298 (4.954) Cm (279:306)
1: SIR of 6 Channels ES-
4.72e3
4-O-caffeoylquinic acid (m/z, 353)
353.00
154.00
193.00
301.00
321.00
515.00
m/z
HO
OH
OH
OH
O
O
O
OH
OH

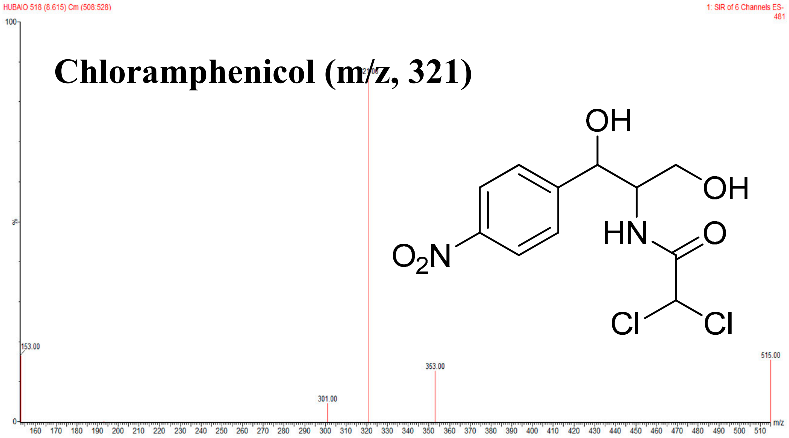
HUBAIO 518 (8.615) Cm (508:528)
1: SIR of 6 Channels ES-
481
Chloramphenicol (m/z, 321)
153.00
301.00
353.00
515.00
m/z
OH
OH
HN
O
Cl
Cl
O2N

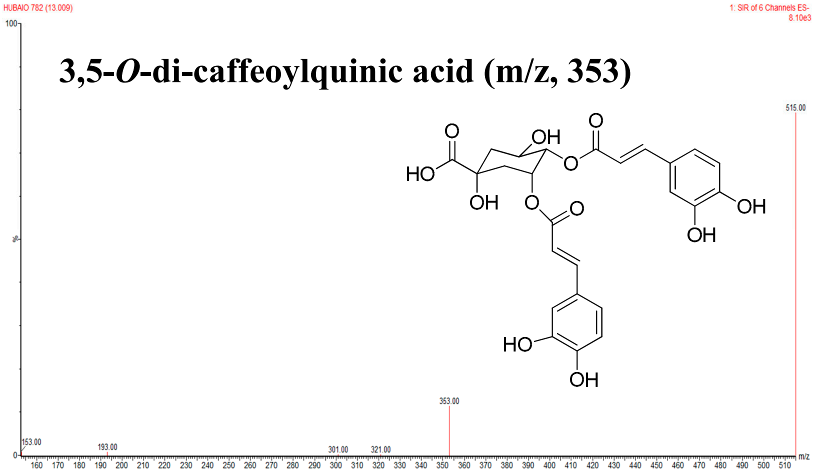
HUBAIO 782 (13.009)
1: SIR of 6 Channels ES-
8.10e3
3,5-O-di-caffeoylquinic acid (m/z, 353)
515.00
353.00
153.00
193.00
301.00
321.00
m/z
HO
OH
OH
O
O
O
O
OH
OH
HO
OH

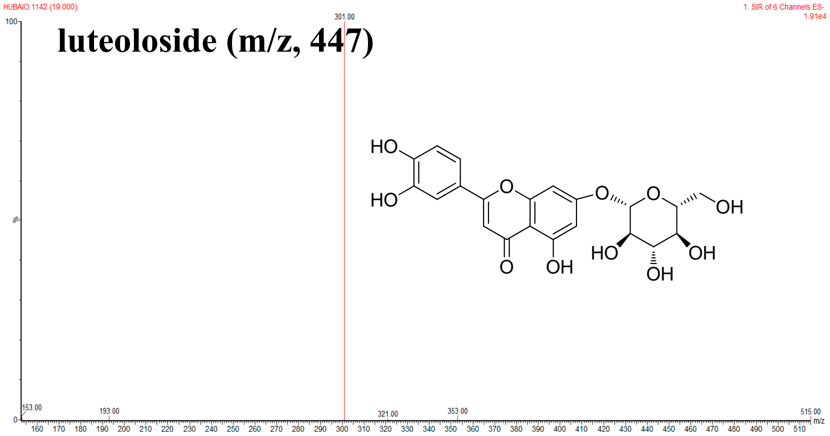
HUBAIO 1142 (19.000)
1: SIR of 6 Channels ES-
1.91e4
luteoloside (m/z, 447)
301.00
153.00
193.00
321.00
353.00
515.00
m/z
HO
HO
O
O
OH
O
O
OH
HO
OH
OH

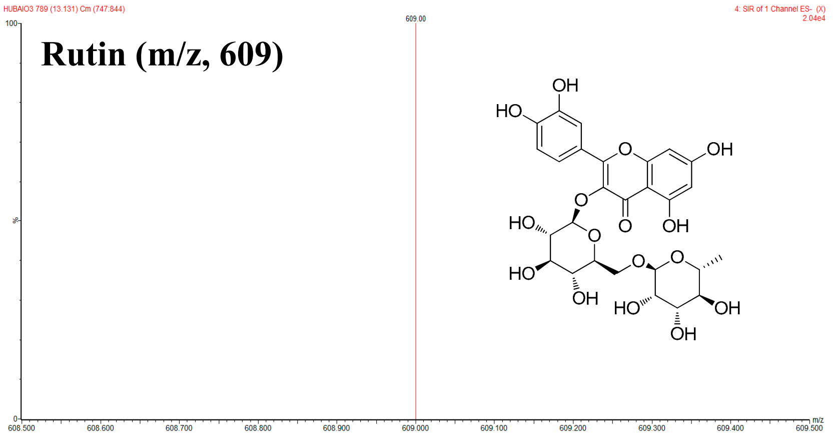

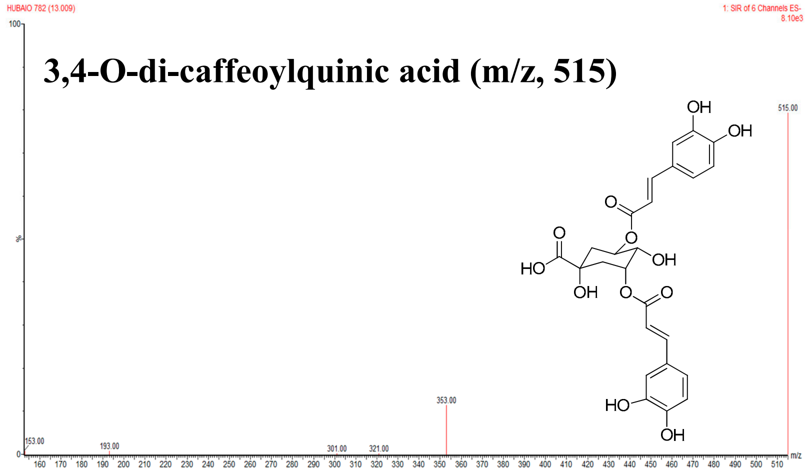

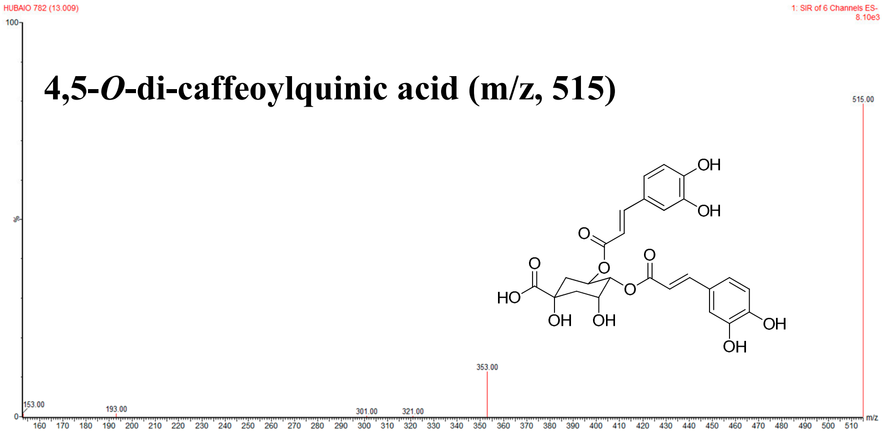

Figure.S1. Chemical structures of 10 analytes and IS.
